# Somatic mutational profiles and germline polygenic risk scores in human cancer

**DOI:** 10.1186/s13073-022-01016-y

**Published:** 2022-02-11

**Authors:** Yuxi Liu, Alexander Gusev, Yujing J. Heng, Ludmil B. Alexandrov, Peter Kraft

**Affiliations:** 1 Department of Epidemiology, Harvard T.H. Chan School of Public Health, Boston, MA 02115 USA; 2 Program in Genetic Epidemiology and Statistical Genetics, Harvard T.H. Chan School of Public Health, 655 Huntington Avenue, Boston, MA 02115 USA; 3 Department of Medical Oncology, Dana-Farber Cancer Institute and Harvard Medical School, Boston, MA 02215 USA; 4 Department of Pathology, Beth Israel Deaconess Medical Center, Harvard Medical School, Boston, MA 02215 USA; 5 Department of Cellular and Molecular Medicine, University of California San Diego, La Jolla, CA 92093 USA; 6 Department of Biostatistics, Harvard T.H. Chan School of Public Health, Boston, MA 02115 USA

**Keywords:** Somatic mutation, Mutational signature, Single-base substitution signature, Polygenic risk score, Cancer

## Abstract

**Background:**

The mutational profile of cancer reflects the activity of the mutagenic processes which have been operative throughout the lineage of the cancer cell. These processes leave characteristic profiles of somatic mutations called mutational signatures. Mutational signatures, including single-base substitution (SBS) signatures, may reflect the effects of exogenous or endogenous exposures.

**Methods:**

We used polygenic risk scores (PRS) to summarize common germline variation associated with cancer risk and other cancer-related traits and examined the association between somatic mutational profiles and germline PRS in 12 cancer types from The Cancer Genome Atlas. Somatic mutational profiles were constructed from whole-exome sequencing data of primary tumors. PRS were calculated for the 12 selected cancer types and 9 non-cancer traits, including cancer risk determinants, hormonal factors, and immune-mediated inflammatory diseases, using germline genetic data and published summary statistics from genome-wide association studies.

**Results:**

We found 17 statistically significant associations between somatic mutational profiles and germline PRS after Bonferroni correction (*p* < 3.15 × 10^−5^), including positive associations between germline inflammatory bowel disease PRS and number of somatic mutations attributed to signature SBS1 in prostate cancer and APOBEC-related signatures in breast cancer. Positive associations were also found between age at menarche PRS and mutation counts of SBS1 in overall and estrogen receptor-positive breast cancer. Consistent with prior studies that found an inverse association between the pubertal development PRS and risk of prostate cancer, likely reflecting hormone-related mechanisms, we found an inverse association between age at menarche PRS and mutation counts of SBS1 in prostate cancer. Inverse associations were also found between several cancer PRS and tumor mutation counts.

**Conclusions:**

Our analysis suggests that there are robust associations between tumor somatic mutational profiles and germline PRS. These may reflect the mechanisms through hormone regulation and immune responses that contribute to cancer etiology and drive cancer progression.

**Supplementary Information:**

The online version contains supplementary material available at 10.1186/s13073-022-01016-y.

## Background

Cancer is driven by the accumulation of somatic mutations. In contrast to germline variants, which are inherited from egg or sperm and occur in the DNA of every cell in the body, somatic mutations are generated from mutational processes of exogenous and endogenous exposures as well as DNA enzymatic modifications and failure/infidelity of DNA repair and replication [1–3]. Mutational processes result in different mutation types (e.g., C>T substitution at the mutated base of ACG motif) with characteristic combinations of mutation types constituting different mutational signatures [1, 4]. Previous studies have identified and confirmed more than 50 distinct signatures of single-base substitution (SBS) derived from the analysis of whole-genome and whole-exome sequences (WES) of multiple cancer types [1, 4–9], but the etiologies for many of these signatures remain largely unexplored. In addition, tumor mutational burden (TMB), which quantifies the total mutations per megabase in a tumor tissue, has been suggested as a biomarker to predict the response of a patient to immunotherapy [10–12]. Recently, The US Food and Drug Administration approved the use of pembrolizumab, a humanized antibody for cancer immunotherapy, in patients with TMB-high solid tumors [13]. However, currently, it is not fully understood the reason most patients with high TMB benefit from immunotherapy.

Mutational signatures reflect the activity of the mutational processes that have been active throughout a person’s life [2]. The identified SBS signatures reflect both processes commonly found across cancer types as well as processes confined to a particular cancer type. For example, signature SBS2 and SBS13, both attributed to the enzymatic activity of the APOBEC family of cytidine deaminases, are present in multiple cancer types [6]. In contrast, signature SBS12, whose etiology is still unknown, is almost exclusively found in liver cancers [1]. Some signatures reflect the effects of lifestyle choices, such as signature SBS4, which is associated with tobacco smoking in multiple cancer types, or environmental exposures, such as signatures SBS7a/b/c/d which are imprinted by exposure to ultraviolet light [1, 14]. Some signatures are caused by endogenous exposures, for example, the clock-like signature SBS1 is attributed to endogenous deamination of 5-methylcytosine [15]. However, the etiologies for many other recently identified signatures remain unclear. Linking those signatures of unknown origin to cancer risk factors may suggest mechanisms and provide avenues for further investigation.

Previous studies of multiple cancer types have found associations between somatic mutational burden and germline genetic variations [16]. For example, germline *MC1R* R alleles carrier status is significantly associated with somatic mutational burden in melanomas [17]. Germline and somatic statuses of *ZNF750* and *CDC27* have an impact on somatic mutational signatures in esophageal squamous cell carcinomas [18]. rs2588809 carrier status in gene *RAD51B* is significantly associated with total somatic mutation counts in breast cancer [19]. rs17000526, a variant associated with *APOBEC3B* expression, is strongly associated with APOBEC signature mutations in bladder cancer [20]. The minor allele (C allele) of germline variant rs12628403 at 22q13.1 has been found to reduce APOBEC3B-like mutagenesis in cancer types with low APOBEC mutations and increase APOBEC3A-like mutagenesis in cancer types with high APOBEC mutations; this variant is a proxy for a 30-kb APOBEC3B-eliminating deletion that is known to increase breast cancer risk as well as APOBEC mutagenesis in breast tumor [7, 20]. Another variant rs2142833 is associated with APOBEC3B-like mutagenesis across cancer types [7]. Carter et al. [21] investigated the interaction between germline variants and somatic events in cancer genes and found robust associations. A recent study by Sun et al. showed that about 13% of the variation in pan-cancer TMB can be explained by common germline genetic variants [22]. In addition to studying the association at the level of individual variants, one study [19] also examined the relationship between polygenic risk scores (PRS), which combine the effect of multiple germline variants, and somatic mutational burden. They found that germline PRS of breast cancer was inversely associated with total somatic mutation counts in breast tumor samples, but the underlying mechanism is still obscure.

Here, we performed a pan-cancer analysis of the association between tumor somatic mutational profiles and germline PRS of cancers and non-cancer traits using data from The Cancer Genome Atlas (TCGA). Studies with comprehensive somatic mutation data do not always have complete and accurate epidemiological exposure data. By aggregating information across individual genetic variants associated with exogenous and endogenous risk factors, as well as other cancers and diseases that may share common biological mechanisms, PRS may increase power to detect associations between germline and somatic variation. Studying the relationship between germline PRS and somatic mutations can also provide insight into the underlying biological mechanisms of cancer development.

## Methods

### Study population

**Fig. 1:**
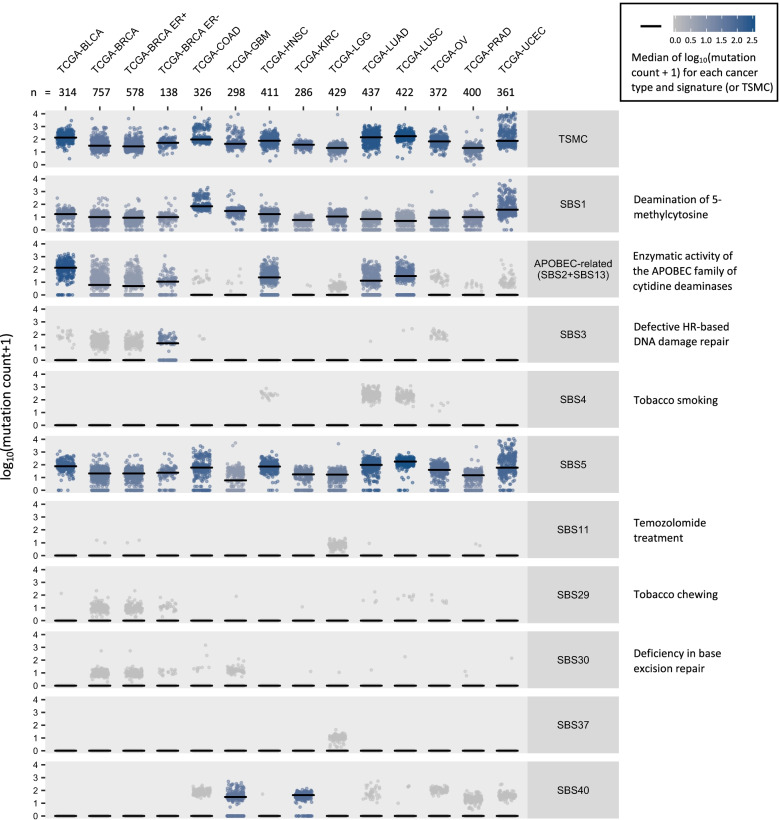
Mutation counts of SBS signatures and TSMC across 12 TCGA cancer types and two subtypes of breast cancer. Each dot represents a tumor sample. The median of log_10_(mutation count + 1) for each cancer type (or subtype) and SBS signature (or TSMC) is represented by both the color of the dots and the short black line in each panel. The number of TCGA samples for each cancer type is shown on the top

TCGA is a joint cancer genomics program of the National Cancer Institute and National Human Genome Research Institute that began in 2006. Over the past decade, TCGA collected more than 20,000 primary cancer and matched normal samples from over 11,000 cases across 33 cancer types [23]. All TCGA biospecimens, including blood and tissue, were collected by their Tissue Source Sites from eligible cancer patients along with their clinical metadata. Genomic data were generated from genomic characterization and high-throughput sequencing of the molecular analytes and were made available to the research community [24]. Here, we selected cancer types based on the total number of cases in TCGA and the availability of large genome-wide association studies (GWAS) for calculating PRS. Twelve cancer types (Fig. 1) with more than 300 patients of European ancestry in TCGA were selected to ensure 80% power (at a type I error rate of 5%) to detect an association between PRS and total somatic mutation counts (TSMC) of similar or greater magnitude as that previously reported between rs2588809 and TSMC [19] (Additional file 1: Figure S1). The initial total sample size was 5296 with the sample size for each cancer type ranging from 314 (bladder urothelial carcinoma) to 802 (breast invasive carcinoma). We further restricted sample type to primary tumor and excluded male cases for breast cancer. Age at cancer diagnosis, sex, and tumor stage were retrieved from the Genomic Data Commons (GDC) data portal (https://portal.gdc.cancer.gov/) [25]. Tumor stage was not available for prostate cancer, endometrial cancer, lower grade glioma, glioblastoma, and ovarian cancer. Samples with missing age at cancer diagnosis, tumor stage (if available), and sex information were further excluded. The remaining 4813 samples with both germline and somatic data available were included in the final analysis. About 8% of TCGA cases carry pathogenic or likely pathogenic germline variants, which may affect somatic mutations [26]. We therefore further identified carriers of pathogenic or likely pathogenic variants as reported by Huang et al. among the selected TCGA cases [26].

### Mutational signatures

Mutational signatures were identified from TCGA WES data using methods based on nonnegative matrix factorization [1]. We obtained the mutation counts for 54 distinct SBS signatures for each selected tumor sample from the ICGC data portal (https://dcc.icgc.org/releases/PCAWG) [7]. In addition, we created a new signature variable called APOBEC-related signatures by summing up the somatic mutation counts of SBS2 and SBS13 (both attributed to APOBEC activity). We analyzed all SBS signatures that were present in more than 20 samples of at least one cancer and in more than 20% of samples of that cancer type. In addition to the SBS signature-specific mutations, we also retrieved TSMC, defined as the total number of somatic missense mutations, for each sample using the maftools R package [27]. Many colorectal and endometrial cancers exhibit genetic hypermutability due to impaired mismatch repair [28–30]. We defined colorectal and endometrial cancer tumors with TSMC greater than 500 to be hypermutable.

### Germline variant data

Raw germline single nucleotide polymorphism (SNP) array data were downloaded from the GDC Legacy Archive (https://portal.gdc.cancer.gov/legacy-archive) [25]. Genotype quality control (QC) was applied to remove variants with > 5% missing calls, Hardy-Weinberg equilibrium *p* < 5 × 10^−6^, or minor allele frequency (MAF) < 1%. To restrict to individuals of European ancestry, genetic principal components (PCs) were computed using post-QC, linkage disequilibrium (LD) pruned variants, and any outlier sample > 6 standard deviations away from the mean along either of the top 2 PCs was removed. All remaining samples and genotypes were then imputed to the Haplotype Reference Consortium reference panel [31].

### Calculation of PRS

The PRS of a trait for subject *i* was calculated as:

$${\mathrm{PRS}}_i=\sum \limits_j{\beta}_j{G}_{ij}$$

where the weight *β*_*j*_ is the log odds ratio (or the beta coefficient for continuous traits) of the trait comparing effect allele to other alleles at SNP *j*, and *G*_*ij*_ is the expected number of effect allele at SNP *j* for subject *i* (allele dosage).

We calculated germline PRS for 23 cancers and non-cancer traits, including the 12 selected cancer types, breast cancer stratified into estrogen receptor-positive (ER+) and estrogen receptor-negative (ER−) subtypes, cancer risk determinants (cigarettes per day, drink per week, and body mass index (BMI)), hormonal factors (age at menarche and age at natural menopause), and immune-mediated inflammatory diseases (inflammatory bowel disease (IBD), ulcerative colitis (UC), Crohn’s disease (CD), and rheumatoid arthritis (RA)) using the TCGA germline variant data and published GWAS summary statistics. For cancer PRS, the list of SNPs and corresponding weights were obtained from one of the four sources: (i) GWAS or PRS paper, (ii) NHGRI-EBI GWAS Catalog [32], (iii) Cancer PRSweb [33], and (iv) The Polygenic Score (PGS) Catalog [34]. For non-cancer PRS, the summary statistics were from GWAS or PRS papers. Sources of GWAS summary statistics for each trait are summarized in Additional file 1: Table S1.

We filtered the SNP list for each trait using different strategies. For those from GWAS Catalog, we removed results from cross-cancer, subgroup, or interaction analysis and restricted to European ancestry studies with a minimum of 2000 cases and 2000 controls. We removed SNPs with a *p* value above the genome-wide significance threshold (*p* > 5 × 10^−8^) for those from GWAS Catalog or GWAS papers. For SNPs from Cancer PRSweb, we used the *p* value threshold with the best performance as evaluated by Nagelkerke’s pseudo-*R*^2^. We did not additionally filter by *p* value nor perform LD clumping on SNPs from PRS papers or PGS Catalog. LD clumping was performed on SNP lists from GWAS paper and GWAS Catalog: we removed SNPs with MAF < 1% or in LD (*r*^2^ > 0.1) with SNPs of smaller *p* value. We used PLINK 2.0 [35] to calculate PRS for each trait and subject from the final list of SNPs (Additional file 2: Table S2).

### Validation of PRS

We evaluated the ability of the calculated PRS to discriminate between specific cancer cases and controls in our data. For each cancer type and PRS, an unadjusted logistic model was fit by treating all patients of that cancer type as cases and a randomly selected subset (with the same sample size as cases) of other cancer cases as controls. The performance of PRS was assessed by the area under the receiver operating characteristic curve. In addition to the 12 cancer types and the two breast cancer subtypes, we also evaluated the performance of PRS on discriminating lung cancer cases combined (lung adenocarcinoma and squamous cell carcinoma) and glioma cases combined (lower grade glioma and glioblastoma). For those cancer types with subtypes or other closely related types (e.g., breast cancer), none of the patients of the other subtypes (or related types) was included as controls when treating one subtype (or type) as cases.

### Association between somatic mutations and germline PRS

We fit a zero-inflated negative binomial model, negative binomial model, or linear model regressing tumor somatic mutation counts on germline cancer or non-cancer PRS for each combination of cancer type (or subtype), SBS signature (or TSMC), and PRS, adjusting for sex (if applicable), age at cancer diagnosis (in years), and the top 10 genetic PCs. TSMC were Winsorized to 98%, where the counts below the first percentile were set to the first percentile and the counts above the 99th percentile were set to the 99th percentile. PRS were standardized to have a mean zero and unit standard deviation before running each model. For each cancer type and SBS signature (or TSMC), a zero-inflated negative binomial model was fit if the proportion of zero-count samples is greater than 10%; otherwise, we fit a negative binomial model directly. If the zero-inflated negative binomial model failed to converge and the proportion of zero-count samples is less than 50% (if greater than or equal to 50%, all models were considered as failed), we tried the negative binomial model. If both two models failed, we transformed the mutation count to log_10_ (count + 1) and fit the linear model. In each attempt, we ran both models with and without PRS (models were considered as failed if either model failed) and obtained *p* value from the likelihood ratio test on these two models. We performed a fixed-effect meta-analysis of the associations between TSMC or each SBS signature and each PRS across cancers. Stouffer’s *Z*-score method was also used to combine the individual *p* values from each cancer type. The *Z*-score for the overall meta-analysis is:

$$Z\sim \frac{\sum_{i=1}^k{w}_i{Z}_i}{\sqrt{\sum_{i=1}^k{w}_i^2}}$$

where *Z*_*i*_ is the *Z*-score for cancer type *i*, *w*_*i*_ is the sample size of cancer type *i*, and *k* is the total number of cancer types and subtypes.

### Sensitivity analysis

To assess the impact of age at cancer diagnosis on the association of germline PRS and somatic mutational burden, we performed association tests without adjusting for age at cancer diagnosis. Spearman correlations were calculated for (i) somatic mutation count and age at cancer diagnosis for each cancer type and signature (or TSMC) and (ii) PRS and age at cancer diagnosis for each cancer type. Tumor stage was only available for some cancers; we performed association tests adjusting for tumor stage for those cancer types. We further adjusted for the hypermutable subtype indicator in models of colorectal and endometrial cancer. The impact of pathogenic variant carrier status on the associations was assessed by running models without PRS and models with PRS and an interaction term of PRS and pathogenic variant carrier status. We obtained 2 d.f. *p* values for the association of PRS and mutational signature from the likelihood ratio test comparing these two models. We further adjusted for the known germline *APOBEC3* risk variants, rs17000526, rs12628403, and rs2142833 [7, 20], separately, in the associations with the APOBEC-related signatures. PRS SNPs in the main analysis underwent stringent filtrations on *p* value (e.g., *p* < 5 × 10^−8^); we calculated another set of PRS for BMI, IBD, and drinks per week using large sets of genome-wide SNPs with weak restriction on *p* value (e.g., *p* < 0.05) from PGS Catalog (Additional file 1: Table S1) and refit the models. All sensitivity analysis models were of the same model types as the models in the main analysis.

## Results

### TCGA germline and somatic data

We included 4813 patients of European ancestry across 12 cancer types with germline variant and somatic mutation data available from TCGA. Overall, somatic data were retrieved for (Fig. 1): (i) TSMC, (ii) nine individual SBS mutational signatures: SBS1, SBS3, SBS4, SBS5, SBS11, SBS29, SBS30, SBS37, and SBS40, among which SBS1, SBS3, SBS30 were attributed to endogenous mutational process (spontaneous deamination of 5-methylcytosine, defective homologous recombination DNA damage repair, and defective DNA base excision repair, respectively); SBS4, SBS11, and SBS29 were attributed to exogenous exposures (tobacco smoking, temozolomide treatment, and tobacco chewing, respectively); other signatures were of unknown etiology, and (iii) one combined APOBEC-related signatures (SBS2 and SBS13). The germline PRS validation results are summarized in Additional file 2: Table S3; all cancer PRS were positively associated with the corresponding cancer case status in the TCGA samples.

### Correlations with age at cancer diagnosis

**Fig. 2:**
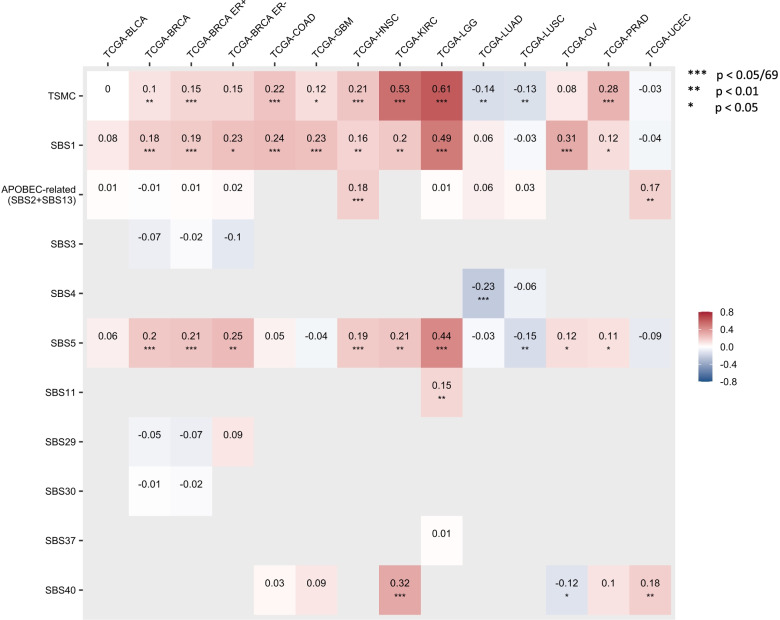
Correlations between somatic mutation counts and age at cancer diagnosis for each cancer type. Only the correlations with age at cancer diagnosis for those cancer-signature pairs included in the analyses are shown in this figure. The number in each cell and the cell color represent the Spearman correlation (*ρ*) between mutation counts of a SBS signature or TSMC (*y*-axis) and age at diagnosis in a cancer type (*x*-axis). Corrections passed the Bonferroni threshold (*p* < 0.05/69 = 7.25 × 10^-4^) are marked with triple asterisk (***), correlations with *p* < 0.01 are marked with double asterisk (**), and correlations with *p* < 0.05 are marked with single asterisk (*)

We assessed the correlations between somatic mutations by signature (or TSMC) and age at cancer diagnosis for each selected cancer type (Fig. 2). We used Bonferroni correction accounting for 69 tests (*p* = 7.25 × 10^−4^), which is the total number of cancer-signature pairs included in the analyses. Consistent with previous studies [5], SBS1 and SBS5, the two clock-like signatures for which the numbers of mutations increase with age, showed strong positive correlations with age at diagnosis for most cancer types, though there exists heterogeneity across cancers. We also evaluated the correlation between the calculated germline PRS and age at diagnosis for each cancer type (Additional file 1: Figure S2). Although none of these associations passed the Bonferroni-adjusted significance threshold accounting for 322 tests (*p* = 1.55 × 10^−4^), the Spearman’s *ρ* with age for most cancer PRS were negative among the cases of that specific cancer, indicating that higher germline PRS of a cancer is associated with earlier diagnosis of that cancer.

### Associations between somatic mutations and germline PRS

Models regressing tumor somatic mutation counts on germline cancer or non-cancer PRS were fit for each combination of cancer type (or subtype), SBS signature (or TSMC), and PRS. The *p* value threshold for significance was 3.15 × 10^−5^, adjusting for multiple comparisons using Bonferroni correction (1587 tests). We found 17 statistically significant associations (Table 1). Significant associations were found for prostate cancer, breast cancer, colorectal cancer, endometrial cancer, and glioblastoma; most of them involved somatic mutation count of SBS1 (deamination of 5-methylcytosine) and the immune-mediated inflammatory diseases (IBD, CD, and UC) PRS. The full association results are in Additional file 2: Table S4. The association results across the 12 cancer types and two breast cancer subtypes for significant signature-PRS pairs are shown in Additional file 1: Figure S3. The association results for significant cancer-PRS pairs across mutational signatures are shown in Additional file 1: Figure S4. Given that there were strong positive correlations between the number of mutations of SBS1 and age at cancer diagnosis in breast cancer (Spearman’s *ρ* = 0.18, *p* = 2.50 × 10^−6^), in colorectal cancer (Spearman’s *ρ* = 0.24, *p* = 2.85 × 10^−5^), and in glioblastoma (Spearman’s *ρ* = 0.23, *p* = 1.11 × 10^−4^); a nominally significant correlation between SBS1 and age in prostate cancer (Spearman’s *ρ* = 0.12, *p* = 0.02); and a significant correlation between SBS40 and age in endometrial cancer (Spearman’s *ρ* = 0.18, *p* = 9.67 × 10^−4^), we further evaluated the associations of somatic mutations and PRS without adjusting for age at cancer diagnosis. There was no substantial change on the top findings, though we observed more significant associations with SBS1 and SBS5, which were likely to be driven by their correlations with age at diagnosis (Additional file 2: Table S5). The inverse association between SBS1 and cigarettes-per-day PRS in colorectal tumor was slightly above the Bonferroni threshold without adjusting for age (*p* = 3.71 × 10^−5^). There was no substantial change of the directions and effect sizes of the significant associations after adjusting for tumor stage where available, though the inverse association between head and neck cancer PRS and TSMC in colorectal tumor became non-significant (Additional file 2: Table S6). After adjusting for hypermutable status for colorectal and endometrial cancer associations, the colorectal cancer associations for both SBS1 with cigarettes-per-day PRS and TSMC with head and neck cancer PRS became non-significant, although the direction of associations remained consistent (with the exception of head and neck cancer PRS and colorectal cancer TSMC; Additional file 2: Table S7). After adjusting for pathogenic variant carrier status, five of the seventeen associations became non-significant (Additional file 2: Table S8). Finally, when we simultaneously adjusted for tumor stage, hypermutable status for colorectal and endometrial cancer, and pathogenic variant carrier status, six associations became non-significant, although, again, the direction of association remained consistent (with the exception of head and neck cancer PRS and colorectal cancer TSMC; Additional file 2: Table S9).

**Table 1:** Significant associations between tumor somatic mutation counts and germline PRS

Cancer type	Somatic mutation count	Germline PRS^a^	Direction of association^b^	*p* value^c^
PRAD	SBS1	Age at menarche	−	2.49 × 10^−9^
PRAD	SBS1	IBD	+	9.04 × 10^−6^
PRAD	SBS1	CD	+	4.63 × 10^−8^
PRAD	SBS1	UC	+	3.71 × 10^−9^
PRAD	SBS1	GBM	−	3.29 × 10^−8^
PRAD	SBS1	HNSC	−	2.07 × 10^−9^
PRAD	SBS1	BMI	+	6.09 × 10^−8^
PRAD	SBS1	Drinks per week	−	1.69 × 10^−5^
BRCA	APOBEC-related^d^	IBD	+	1.79 × 10^−6^
BRCA	SBS1	Age at menarche	+	1.47 × 10^−5^
BRCA ER+	SBS1	Age at menarche	+	2.89 × 10^−5^
BRCA ER-	SBS1	HNSC	−	1.99 × 10^−6^
BRCA ER-	TSMC	HNSC	−	5.31 × 10^−7^
COAD	SBS1	Cigarettes per day	−	1.71 × 10^−5^
COAD	TSMC	HNSC	−	1.71 × 10^−5^
GBM	SBS1	OV	+	1.97 × 10^−5^
UCEC	SBS40	CD	−	7.71 × 10^−7^

^a^PRS is calculated from the germline genetic data of the same TCGA patient as the tumor sample

^b^Direction of the association between somatic mutation count and PRS from zero-inflated negative binomial model, negative binomial model, or linear model adjusting for age at cancer diagnosis, sex, and the top 10 genetic PCs

^c^*P* value associated with PRS. *P* values are obtained from likelihood ratio test of model with PRS and model without PRS. For zero-inflated negative binomial model, the results are from testing the count and logistic model jointly. Age at cancer diagnosis, sex, and the top 10 genetic PCs were adjusted as covariates in all models

^d^APOBEC-related signature count is the sum of SBS2 and SBS13 mutation counts, both signatures are attributed to the enzymatic activity of the APOBEC family of cytidine deaminases

Consistent with a previous study [19], which found a significant inverse association between breast cancer PRS and TSMC in breast tumor samples for ER+ patients, we found inverse associations between the somatic mutation counts of APOBEC-related signatures and breast cancer PRS (both overall and ER+ specific) in breast tumors, though the associations were slightly above the significance threshold (*p* = 5.08 × 10^−5^ for ER+, *p* = 1.14 × 10^−4^ for overall). We further adjusted for the known germline *APOBEC3* risk variants, rs17000526, rs12628403, and rs2142833, in the associations with the APOBEC-related signatures. rs17000526 is in LD with bladder cancer PRS SNP rs1014971 (*r* ^2^ = 0.98) and breast cancer PRS SNP rs5750715 (*r* ^2^ = 0.16); rs12628403 is also in LD with rs5750715 (*r* ^2^ = 0.18); rs2142833 was not included in the calculation of any cancer or non-cancer PRS or in LD (*r* ^2^ > 0.1) with any PRS variants. There was no substantial change in the top associations with the APOBEC-related signatures after adjusting for these variants separately. We observed an additional significant inverse association between age at menarche PRS and APOBEC-related signatures in breast tumor (*p* = 2.57 × 10^−5^) and a significant positive association for CD PRS (*p* = 2.75 × 10^−5^) after adjusting for rs2142833. Consistent with a prior study [20], we observed a significant association between rs17000526 and the APOBEC-related signatures in bladder tumors (regression coefficient = 0.21 for the rs17000526-A allele, *p* = 1.81 × 10^−4^). This association was not significant in the overall breast tumors. However, we did find a nominally significant association between rs17000526 and the APOBEC-related signatures in ER+ breast tumors (coefficient = 0.10, *p* = 0.04). In addition, significant associations were also found for rs12628403 in the overall (regression coefficient for rs12628403-A allele = − 0.25, *p* = 1.59 × 10^−3^) and ER+ breast tumors (coefficient = − 0.31, *p* = 1.16 × 10^−3^).

Except for breast cancer, the most significant association of somatic mutations and germline PRS for the same cancer type was observed between prostate cancer PRS and SBS1 count in prostate tumor (*p* = 7.87 × 10^−5^). We did not see any significant association between mutation counts in lung tumor and cigarettes-per-day PRS (smallest *p* values: *p* = 3.32 × 10^−3^ for TSMC in lung adenocarcinoma; *p* = 0.03 for SBS1 in lung squamous cell carcinoma). Age at natural menopause and RA PRS were also not significantly associated with mutation counts in any cancer type (smallest *p* values: *p* = 1.96 × 10^−4^ for age at natural menopause PRS and SBS5 count in prostate tumor; *p* = 9.32 × 10^−5^ for RA and TSMC in glioblastoma).

Consistent with the main analysis, we observed a significant inverse association between drinks-per-week PRS calculated using genome-wide SNPs (i.e., including SNPs that do not meet the genome-wide significance threshold) and SBS1 count in prostate tumor (*p* = 6.35 × 10^−7^). Positive associations between BMI PRS and SBS1 count in prostate tumor (*p* = 2.34 × 10^−3^), IBD PRS and SBS1 count in prostate tumor (*p* = 0.20), and IBD PRS and APOBEC mutation count in breast tumor (*p* = 0.22) all became non-significant using genome-wide SNPs.

**Fig. 3:**
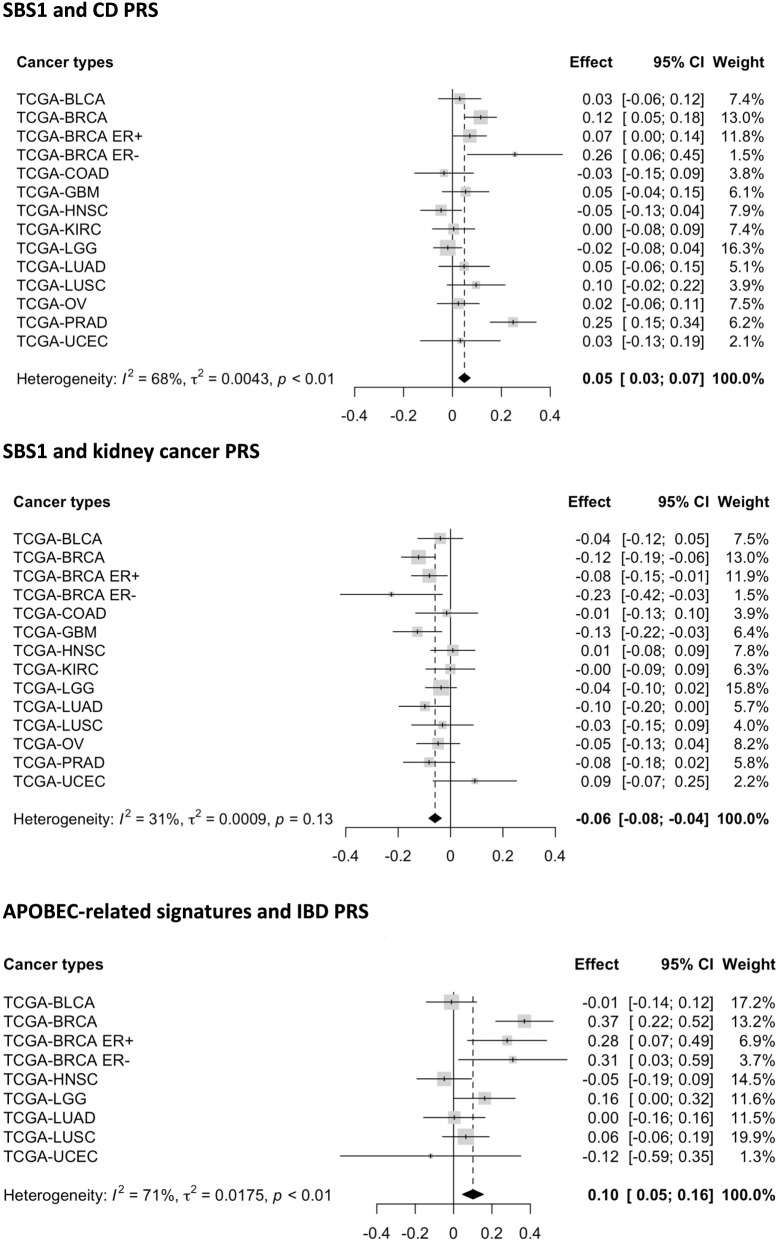
Significant association results from meta-analyses. The fixed-effect and Stouffer’s *p* values for the association between SBS1 and CD PRS are *p* = 4.29 × 10^−5^ and *p* = 1.33 × 10^−5^; for the association between SBS1 and the kidney cancer PRS, they are *p* = 9.05 × 10^−7^ and *p* = 2.10 × 10^−4^, and for the association between APOBEC-related signatures and IBD PRS, they are *p* = 2.61 × 10^−4^ and *p* = 3.13 × 10^−4^. There are significant heterogeneities in the effect sizes for the associations between SBS1 and CD PRS and APOBEC-related signatures and IBD PRS across cancers (*p* < 0.01). The effect sizes and 95% CI for PRS are plotted using gray squares and black horizontal lines. The size of the gray squares represents the weight in the fixed-effect model for each cancer type. Dashed line and diamond represent the results from the fixed-effect model

For the meta-analyses of signature-PRS associations across cancers, we used Bonferroni correction to adjust for multiple comparisons of 115 tests, which is the total number of signature-PRS pairs, excluding those only available for breast cancer (overall, ER+, and ER−) and lung cancer (adenocarcinoma and squamous cell carcinoma). The associations of SBS1 with CD PRS, SBS1 with kidney cancer PRS, and APOBEC-related signatures with IBD PRS were significant using both the fixed-effect model and the Stouffer’s *Z* method (*p* < 4.35 × 10^−4^) (Fig. 3). Overall, we found 19 associations that were statistically significant based on either the fixed-effect model or Stouffer's method (Additional file 2: Table S10).

## Discussion

We performed a comprehensive analysis on the association between tumor somatic mutational profiles and germline PRS for various cancers and non-cancer traits, leveraging mutational signatures and germline variant data of 12 cancer types, as well as summary statistics from recent large GWAS. Our results demonstrate that there are robust associations between somatic mutational profiles and germline PRS in human cancer. Some of these PRS reflect genetic variations underlying cancer-related risk factors. Linking mutational signatures to these exogenous and endogenous risk factors represented by PRS may suggest the etiology of associated mutational processes. Other PRS reflect the germline genetic contribution to cancer risk. Studying the relationships of PRS with tumor somatic mutational burden may shed light on the underlying mechanism of cancer development that is attributed to germline-somatic interactions.

**Fig. 4:**
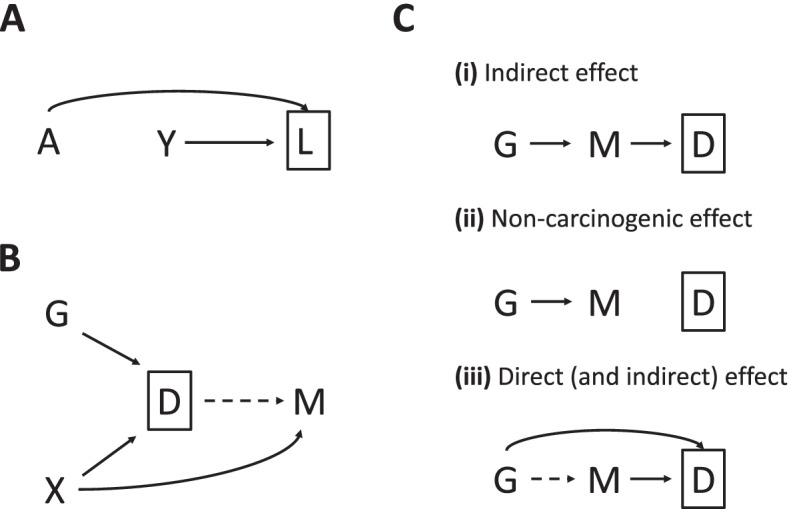
Hypothetical relationships between germline PRS, risk factor, mutational signature, and diagnosis of cancer. **a** Exposure is associated with the outcome due to collider bias. Exposure (*A*) will be associated with outcome (*Y*) within levels of their common effect (*L*) even if there is no causal effect of exposure (*A*) on the outcome (*Y*). **b** Cancer PRS is associated with mutational signature due to collider bias. Cancer diagnosis (*D*) may or may not have an effect on somatic mutations of certain mutational signature (*M*). The cancer risk factor (*X*) is independent of cancer PRS (*G*) in the general population and is also associated with mutational signatures (*M*). Conditioning on diagnosis (*D*, i.e., studying cancer cases only) would induce collider bias on the relationship between cancer PRS (*G*) and mutational signature (*M*). If the cancer risk factor (*X*) is positively associated with both cancer diagnosis (*D*) and mutational signature (*M*), then an inverse association between cancer PRS (*G*) and mutational signature (*M*) is likely to be observed. **c** Three possible relationships between cancer or non-cancer PRS, mutational signature, and cancer diagnosis assuming no reverse causation. (i) Indirect effect: PRS (*G*) has an indirect effect on tumor development and diagnosis (*D*) through inducing somatic mutations of certain mutational signature (*M*); (ii) non-carcinogenic effect: PRS (*G*) has an effect on inducing somatic mutations of certain mutational signature (*M*) but neither PRS (*G*) nor somatic mutations (*M*) has an effect on tumor development and diagnosis (*D*); (iii) direct (and indirect) effect: PRS (*G*) has a direct effect on tumor development and diagnosis (*D*) that is not through the effect of somatic mutations (*M*) and may or may not have an indirect effect through somatic mutations (*M*). In this case, conditioning on diagnosis (*D*, i.e., studying cancer cases only) would induce collider bias on the relationship between PRS (*G*) and mutational signature (*M*)

We found several inverse associations between germline cancer PRS and tumor mutation counts. Interestingly, the overall breast cancer PRS and ER+-specific PRS were both associated with the somatic mutation count of APOBEC-related signatures in breast tumor. These inverse relationships are consistent with a previous study by Zhu et al. [19]. Similarly, Qing et al. [36] reported a significant inverse relationship between germline high-functional-impact variants and somatic mutations in cancer hallmark genes among TCGA patients across age groups. It has been hypothesized that patients with higher germline variant burden tend to develop cancer at a younger age thus would have fewer acquired somatic mutations, whereas patients at a lower germline genetic risk may need longer time for cancer development, which is mainly driven by the accumulation of somatic mutations [19, 36]. However, we observed this inverse relationship for APOBEC-related signatures, which did not show clock-like behavior in previous studies [1, 5]. In our data, there was also no statistically significant correlation between mutation count of APOBEC-related signatures in breast cancer and age at cancer diagnosis (Spearman’s *ρ* = − 0.01, *p* = 0.73, Fig. 2). The direction of this association did not alter in sensitivity analysis models. This inverse relationship may indicate that breast tumors in women with low breast cancer PRS are likely to have higher APOBEC mutation counts (due to yet-to-be-determined biological mechanisms), but it may also be a result of collider bias. Collider bias arises when we condition on a common effect of the exposure and the outcome (Fig. 4a). The exposure and the outcome can be associated in one direction within levels of their common effect even if there is no causal effect of the exposure on the outcome or the causal effect is in the other direction [37]. In our case, if other breast cancer risk factors that are independent of the breast cancer PRS in the general population are positively associated with breast cancer risk and APOBEC mutation counts, then the breast cancer PRS and APOBEC mutation counts could be negatively correlated among cases (Fig. 4b). This negative relationship is supported by findings from Aschard et al. [38], but the direction may not always be negative especially if the association of the risk factor with cancer risk and mutation counts are not both positive. Few studies have directly assessed the associations between established cancer risk factors and SBS signatures. A study reported no impact of BMI, cigarette smoking, and alcohol consumption on APOBEC signatures in TCGA [39].

In another simple setting assuming no reverse causation, there might be three possible causal relationships between cancer or non-cancer PRS, mutational signature, and cancer diagnosis given that a significant association between a germline PRS and a mutational signature is observed (Fig. 4c). Collider bias would also arise if both the PRS and mutational signature have a direct effect (or effect through other pathways) on tumor development and cancer diagnosis. If that is the case, a non-causal association between germline PRS and mutational signature would be observed if we only include cancer cases in the study. This scenario is plausible for breast cancer, as many breast cancer susceptibility variants identified from GWAS are protein-coding variants or directly regulate the expression of cancer genes (e.g., missense variant rs35383942 in *PHLDA3*, which encodes a p53-regulated repressor of Akt [40]), thus have effects on breast cancer development through other functional pathways that are not mediated by the acquirement of somatic mutations [41–44]. To avoid the potential collider biases, somatic mutation profiling needs to be performed on both tumor and normal tissue at the cancer sites of interest and the sampling of the study population should be independent of cancer status. The Human Tumor Atlas Network [45], which seeks to construct comprehensive atlases of molecular and cellular features of cancers, starting from precancerous lesions to advanced disease, could address these questions. In addition to the breast cancer associations, inverse relationships were also observed for other five cancer PRS associations (head and neck cancer PRS and glioblastoma PRS with SBS1 in prostate tumor, head and neck cancer PRS with SBS1 and TSMC in ER− breast tumor, and head and neck cancer PRS with TSMC in colorectal tumor), either adjusting or not adjusting for age at diagnosis. Interestingly, there was a significant inverse association between kidney cancer PRS and SBS1 across cancers (Fig. 3), though none of the individual associations reached the significance threshold (smallest *p* value: *p* = 3.65 × 10^−4^ for breast cancer). These observed inverse associations may reflect shared etiology, but these may also be explained by the collider bias described above.

For non-cancer traits, germline PRS of age at menarche was found to be significantly associated with SBS1 count in both overall and ER+ breast tumor. Specifically, we found that breast cancer patients with higher age at menarche PRS, a surrogate for later menarche, tend to have more SBS1 mutations. It has been well established that early age at menarche is associated with increased breast cancer risk [46, 47]. Therefore, it is possible that breast cancer patients with higher age at menarche PRS thus lower breast cancer risk (most likely but not necessarily) would tend to develop breast cancer at a later age which is mainly driven by the accumulation of somatic mutations. Indeed, we observed this positive association for the two clock-like signatures SBS1 and SBS5, but not for the APOBEC-related signatures (Additional file 1: Figure S4). We also observed a significant association between age at menarche PRS and SBS1 count in prostate tumor. Although males do not exhibit menarche, a previous study reported a strong genetic correlation (*r*_*g*_ = 0.74) between female and male puberty timing, represented by age at menarche and age at voice breaking, respectively [47]. Therefore, we hypothesize that the effect of hormonal factor PRS on the number of somatic mutations in prostate tumors may be explained by the shared regulatory mechanism of puberty timing in men and women. Prostate cancer has long been recognized as a hormone-related cancer; previous studies have reported associations between various hormones (e.g., insulin-like growth factor 1 (IGF-1), testosterone) and the risk of prostate cancer [48–50]. Mendelian randomization studies also reported a consistent protective effect of later pubertal development on prostate cancer risk [47, 51]. All these suggest an etiological relevance between the timing of puberty and incidence of prostate cancer involving shared effect of hormonal factors. Previous findings highlighted the roles of androgen and IGF-1, of which the circulating levels increase dramatically during puberty, in prostate carcinogenesis [51–55]. They proposed that the concentrations of these hormones during this susceptibility window when luminal cells start to appear and the prostate becomes mature may have an impact on prostate cancer risk in later life [56–58]. Our findings suggest a potential pathway through the hormone-related markers of puberty timing on the accumulation of SBS1 mutations in prostate tissue, which may drive tumor development.

Inflammatory bowel disease PRS were found to be associated with somatic mutational profiles in multiple hormone-related cancers (breast, prostate, and endometrial cancer). Previous studies have established CD and UC as risk factors for overall cancer [59–61], but whether these associations are driven by shared genetic susceptibility or other common lifestyle and environmental factors remains unanswered by these observational studies. In the present work, we found a positive association between IBD PRS and somatic mutations of APOBEC-related signatures in breast cancer; this IBD-APOBEC association was broadly significant across cancers (Fig. 3). Prior evidence have shown an increased risk of breast cancer among UC and CD patients, and first-degree relatives of CD patients [61, 62]. Several potential mechanisms have been proposed. Interleukin-1 polymorphism has been linked to the risk of many diseases, including breast cancer and IBD [63–66]. A study [67] identified 53 common differentially expressed genes and the shared Interleukin-17 and NF-κB signaling pathways for breast cancer and CD patients compared with controls. Hovde et al. [61] suggested that the downregulation of breast cancer resistance protein in UC patients may be related to breast cancer etiology. In addition to breast cancer, we also observed robust positive relationships between IBD PRS (IBD, UC, and CD) and somatic mutation count of SBS1 in prostate tumor; the association between CD and SBS1 was broadly significant across the 12 cancer types in our study (Fig. 3). Recent meta-analyses and a large cohort study concluded that patients with IBD have an increased risk of prostate cancer [68–70]; it has been proposed that shared risk alleles may partially explain this association [68, 71]. Folate hydrolase 1 (FOLH1) or prostate-specific membrane antigen (PSMA) is overexpressed in both IBD and prostate cancer [72–74]. Studies have reported that inhibition of FOLH1/PSMA ameliorates IBD symptom in mice models [72] and also leads to tumor regression in preclinical models [75]. Our results suggest there is a link between inherited genetic variants that contribute to the development of inflammatory bowel disease and acquired somatic mutations in these hormonal-related cancers. Future studies need to further investigate the mechanisms underlying the associations between specific PRS and specific SBS signatures, especially between IBD PRS and APOBEC-related signatures and CD PRS and SBS1. One direction might be looking at the associations between these mutational signatures and the measured immune-related markers in TCGA. This may also provide novel insight into the mechanism underlying the association between TMB and benefit from immunotherapy. Interestingly, we found a significant inverse association between CD PRS and SBS40 count in endometrial cancer. There are limited studies looking at the association between IBD and the risk of endometrial cancer. Our results may suggest potential mechanisms underlying SBS40 through inflammatory processes. We did not observe any significant association between IBD PRS and somatic mutation count in colorectal cancer (*p* > 0.05), though having IBD has long been recognized as a risk factor for developing colorectal cancer [76, 77]. It is possible that the observed link between IBD and colorectal cancer risk cannot be explained by any germline-somatic associations studied here, but this may also be a power issue given the small sample size for colorectal cancer.

A significant positive association was observed between BMI PRS and SBS1 count in prostate cancer. Obesity has been established as an independent risk factor for advanced or fatal prostate cancer [78–80]. Studies also have shown that high BMI is associated with a decreased risk of low-grade prostate cancer [78, 79, 81, 82]. However, these associations may be due to diagnostic bias. Men with higher BMI tend to have lower prostate-specific antigen levels [83, 84], which makes them less likely to have a biopsy, and larger prostates, which makes it harder to find the tumor from biopsy [85, 86]. Mendelian randomization studies have found little evidence for a causal relationship between BMI and prostate cancer incidence [87, 88]. Therefore, it is likely that a higher BMI PRS is associated with an older age at diagnosis, and since SBS1 counts increase with age, people with higher BMI PRS would have higher SBS1 counts as well. We did not adjust for tumor stage or grade for prostate cancer cases, as it was unavailable in our data. We adjusted for age at diagnosis, and the BMI PRS is not significantly associated with age at diagnosis of prostate cancers in our study (Additional file 1: Figure S2), but we still observed this strong positive association. Future studies need to further investigate this association with SBS1 incorporating both tumor stage and grade information, and carefully control for the potential biases. Nevertheless, this positive association for BMI may explain the observed inverse association between age at menarche PRS and SBS1 count in prostate tumor. While the latter association might be partially explained by hormonal effect, it might also be explained by the fact that a higher BMI is associated with an earlier puberty [89]. If people with lower age at menarche PRS have higher BMI PRS, then the inverse association of age at menarche and the positive association of BMI with somatic mutations would be expected, but again, these hypotheses need to be confirmed by future studies.

In addition to the associations for non-cancer PRS mentioned above, we also observed an inverse association between drinks-per-week PRS and SBS1 count in prostate tumor. A recent study showed that alcohol intake was inversely associated with lethal prostate cancer [90]. This observed inverse association in our study needs to be further investigated with tumor stage and grade information. Another inverse association was observed between cigarettes-per-day PRS and SBS1 counts in colorectal tumor. However, this association became non-significant after further adjusting for hypermutable status (Additional file 2: Table S6). There exists inconsistency in the association results for BMI and IBD PRS between using genome-wide SNPs and SNPs passed a more stringent *p* value threshold, but this is also expected given that these PRS may capture different effects. Using a small set of top SNPs may reflect more of the mechanism-related genetic effects of a trait compared with using a large set of genome-wide SNPs which may capture a substantial amount of pleiotropic effect. Therefore, we chose to use parsimonious PRS in the main analysis to minimize the impact of pleiotropy and confounding.

Our study has limitations. The calculated PRS only explains a small proportion of the heritability thus may not fully represent the germline genetic variant burden underlying a trait. The association of germline PRS and somatic mutations might be driven by only a few SNPs included in the PRS, while the remaining SNPs may involve in other biological pathways, unrelated to the mutational processes. In particular, associations involving a PRS for an exogenous exposure like drinks-per-week could only be interpreted as compelling evidence for the association between the exposure itself and mutational signatures if the SNPs in the PRS only affect mutational processes thru their effects on exposure [91]. This need not be the case if some SNPs affect multiple biologic pathways (pleiotropy) [92, 93]. However, as discussed above, we used a parsimonious set of SNPs restricted to those exhibiting the strongest associations with the exposure or disease under study, minimizing the risk of pleiotropy. Nevertheless, it would still be of interest for future studies to look at the associations of somatic mutations with genome-wide PRS and compare to the results of using parsimonious sets of SNPs. Another thing to be cautious of when interpreting the results is that the associations were with somatic mutation counts in the developed tumor, but not cancer risk. Although we adjusted for tumor stage for most cancer types, we did not adjust for or perform subgroup analysis by tumor grade, as this information is not available for the selected TCGA patients. Although there was no substantial change of the directions of association or effect estimates in the sensitivity analysis compared to the main analysis, the *p* values of some associations fluctuated up or down the significance threshold depending on the SNPs included in the PRS and the covariates included in the model. Bonferroni correction for multiple testing is conservative; we expect to identify more significant associations with a larger sample size. Our study population was restricted to individuals of European ancestry. We do not have data on country of origin. Given that environmental exposures can vary by geographical regions, which may lead to the different prevalence of certain mutational signatures across regions, we suggest future studies to account for these potential differences.

There are several strengths of our study. We performed a comprehensive pan-cancer analysis of the relationship between 14 cancer PRS and 9 non-cancer PRS, and TSMC as well as the number of mutations attributed to 10 SBS signatures across 12 cancer types. Using PRS for exogenous exposures such as cigarettes-per-day allowed us to examine the association between these exposures (otherwise unmeasured) and mutational signatures, avoiding bias from confounding and reverse causation in traditional observational studies—although concerns regarding pleiotropy mentioned above temper conclusions regarding causal associations between these exposures and mutational signatures. Also, we have a sufficient sample size for detecting associations of similar strength as the previous study [19] in each cancer type with a high power, which may not be achievable using exposure data that usually have missing metadata. We assessed the impact of age at cancer diagnosis, tumor stage, hypermutable status, pathogenic variant carrier status, and established signature-associated variants on the germline-somatic associations. However, it would still be useful to collect and analyze epidemiological data on exposures in future studies for a better understanding of the role of germline genetic variations underlying these associations. Future studies can also look at the association between signature-specific mutation count and immune features, hormonal markers, and expression levels of cancer susceptibility genes to further investigate the underlying mechanisms.

## Conclusions

In conclusion, our findings indicate that there are robust associations between somatic mutational profiles and germline PRS in human cancer. Our results demonstrate evidence for germline-somatic associations between inflammatory bowel disease PRS and somatic mutations (SBS1 and APOBEC-related signatures) in breast cancer and prostate cancer, and between age at menarche PRS and somatic mutations (SBS1) in breast and prostate cancer. Our results are relevant to the etiology of mutational signatures and the underlying biological mechanisms of cancer development.

## Supplementary Information

**Additional file 1 **: **Table S1.** Sources of GWAS summary statistics for calculating PRS, **Figure S1.** Power as a function of proportion of variance in TSMC explained by PRS for various sample sizes, **Figure S2.** Correlations between germline PRS and age at cancer diagnosis for each cancer type, **Figure S3.** Significant associations between SBS signatures (or TSMC) and germline PRS across cancers, **Figure S4.** Significant associations between somatic mutation counts and germline PRS across SBS signatures and TSMC.

**Additional file 2 **: **Table S2.** SNPs used for calculating germline PRS, **Table S3.** PRS validation results, **Table S4.** Full association results of somatic mutations and germline PRS, **Table S5.** Association results without adjusting for age at cancer diagnosis, **Table S6.** Association results adjusting for tumor stage, **Table S7.** Association results adjusting for hypermutable status for colorectal and endometrial cancer, **Table S8.** Association results adjusting for pathogenic variant carrier status, **Table S9.** Association results adjusting for tumor stage, hypermutable status for colorectal and endometrial cancer, and pathogenic variant carrier status. **Table S10.** Meta-analysis results of the somatic mutations and germline PRS associations across cancers.

## Data Availability

The TCGA germline variants data are available at https://portal.gdc.cancer.gov/legacy-archive [25]. The TCGA somatic mutations and mutational signatures data are available from the maftools R package [27] and at https://dcc.icgc.org/releases/PCAWG [7]. The TCGA clinical data are available at https://portal.gdc.cancer.gov/ [25]. The TCGA pathogenic germline variants data are available at https://gdc.cancer.gov/about-data/publications/PanCanAtlas-Germline-AWG [25, 26].

## Abbreviations

BLCA: Bladder urothelial carcinoma
BMI: Body mass index
BRCA: Breast invasive carcinoma
CD: Crohn’s disease
COAD: Colon adenocarcinoma
ER−: Estrogen receptor-negative
ER+: Estrogen receptor-positive
FOLH1: Folate hydrolase 1
GBM: Glioblastoma multiforme
GDC: Genomic Data Commons
GWAS: Genome-wide association studies
HNSC: Head and neck squamous cell carcinoma
IBD: Inflammatory bowel disease
IGF-1: Insulin-like growth factor 1
KIRC: Kidney renal clear cell carcinoma
LD: Linkage disequilibrium
LGG: Brain lower grade glioma
LUAD: Lung adenocarcinoma
LUSC: Lung squamous cell carcinoma
MAF: Minor allele frequency
OV: Ovarian serous cystadenocarcinoma
PCs: Principal components
PRAD: Prostate adenocarcinoma
PGS: Polygenic score
PRS: Polygenic risk scores
PSMA: Prostate-specific membrane antigen
QC: Quality control
RA: Rheumatoid arthritis
SBS: Single-base substitution
SNP: Single nucleotide polymorphism
TCGA: The Cancer Genome Atlas
TMB: Tumor mutational burden
TSMC: Total somatic mutation counts
UC: Ulcerative colitis
UCEC: Uterine corpus endometrial carcinoma
WES: Whole-exome sequences
