# Supplementary material for: Somatic mutational profiles and germline polygenic risk scores in human cancer
Source: Genome Med. 2022 Feb 11;14:14. doi: 10.1186/s13073-022-01016-y (PMC8832866; doi:10.1186/s13073-022-01016-y)
Supplement: Supplementary file 1 — Additional file 1 : Table S1. Sources of GWAS summary statistics for calculating PRS, Figure S1. Power as a function of proportion of variance in TSMC explained by PRS for various sample sizes, Figure S2. Correlations between germline PRS and age at cancer diagnosis for each cancer type, Figure S3. Significant associations between SBS signatures (or TSMC) and germline PRS across cancers, Figure S4. Significant associations between somatic mutation counts and germline PRS across SBS signatures and TSMC. [file 13073_2022_1016_MOESM1_ESM.docx]

**Additional file 1**

**Supplementary Table**

**Table S1.** Sources of GWAS summary statistics for calculating PRS

**Supplementary Figures**

**Figure S1.** Power as a function of proportion of variance in TSMC explained by PRS for various sample sizes

**Figure S2.** Correlations between germline PRS and age at cancer diagnosis for each cancer type

**Figure S3.** Significant associations between SBS signatures (or TSMC) and germline PRS across cancers

**Figure S4.** Significant associations between somatic mutation counts and germline PRS across SBS signatures and TSMC

**Table S1.** Sources of GWAS summary statistics for calculating PRS

|  | **Phenotype** | **Number of SNPs^a^** | **GWAS Source** | **PMID/EFO ID/phecode/Polygenic Score ID^b^** | **Access/Publication Date** |
| --- | --- | --- | --- | --- | --- |
| **Cancer PRS** | BLCA | 15 | Cancer PRSweb (1-8) | 189.2 (cancer of bladder) | 4/23/2020 |
|  | BRCA | 240 | PRS paper (9) | 30554720 | Jan-2019 |
|  | BRCA ER+ | 240 | PRS paper (9) | 30554720 | Jan-2019 |
|  | BRCA ER- | 240 | PRS paper (9) | 30554720 | Jan-2019 |
|  | COAD | 87 | Cancer PRSweb (1, 10) | 153 (colorectal cancer) | 4/23/2020 |
|  | GBM | 5 | Cancer PRSweb (1, 11, 12) | 191.11 (cancer of brain) | 4/23/2020 |
|  | HNSC | 12 | PGS Catalog (13, 14) | PGS000081 | Feb-2021 |
|  | KIRC | 16 | PGS Catalog (13, 14) | PGS000076 | Feb-2021 |
|  | LGG | 19 | GWAS Catalog (12, 15) | EFO_0005543 (glioma) | 5/27/2020 |
|  | LUAD | 15 | GWAS Catalog (15-17) | EFO_0000571 (lung adenocarcinoma) | 5/27/2020 |
|  | LUSC | 6 | GWAS Catalog (15, 17) | EFO_0000708 (squamous cell lung carcinoma) | 5/27/2020 |
|  | OV | 20 | Cancer PRSweb (1, 18) | 184.11 (malignant neoplasm of ovary) | 4/23/2020 |
|  | PRAD | 963 | Cancer PRSweb (1, 19) | 185 (cancer of prostate) | 4/23/2020 |
|  | UCEC | 18 | Cancer PRSweb (1, 20-22) | 182 (malignant neoplasm of uterus) | 4/23/2020 |
| **Non-cancer PRS** | Age at menarche | 321 | GWAS paper (23) | 28436984 | Jun-2017 |
|  | Age at natural menopause | 46 | PRS paper (24) | 27760082 | Feb-2017 |
|  | BMI | 73 | GWAS paper (25) | 27427428 | Jun-2016 |
|  | BMI (genome-wide SNPs)^c^ | 2079140 | PGS Catalog (14, 26) | PGS000027 | Apr-2019 |
|  | Cigarettes per day | 46 | GWAS paper (27) | 30643251 | Feb-2019 |
|  | Drink per week | 89 | GWAS paper (27) | 30643251 | Feb-2019 |
|  | Drink per week (genome-wide SNPs)^c^ | 1110306 | PGS Catalog (14, 28) | PGS000203 | Jun-2020 |
|  | IBD | 151 | GWAS paper (29) | 26192919 | Sep-2019 |
|  | IBD (genome-wide SNPs)^c^ | 6453073 | PGS Catalog (14, 30) | PGS000017 | Sep-2018 |
|  | UC | 85 | GWAS paper (29) | 26192919 | Sep-2019 |
|  | CD | 136 | GWAS paper (29) | 26192919 | Sep-2019 |
|  | RA | 60 | GWAS paper (31) | 24390342 | Feb-2014 |

**^a^** Number of SNPs included in the final calculation of PRS in our study; not necessarily the total number of SNPs from the source due to the filtering steps discussed in the main text and germline data availability. See Additional file: Table S2 for the list of SNPs included in the final calculation for each phenotype.

**^b^** PMID is listed if from PRS/GWAS paper; EFO ID is listed if from GWAS Catalog; phecode is listed if from Cancer PRSweb; Polygenic Score ID is listed if from PGS Catalog.

**^c^** These PRS were calculated using genome-wide SNPs, which were different from the parsimonious PRS used in the main analysis.

**
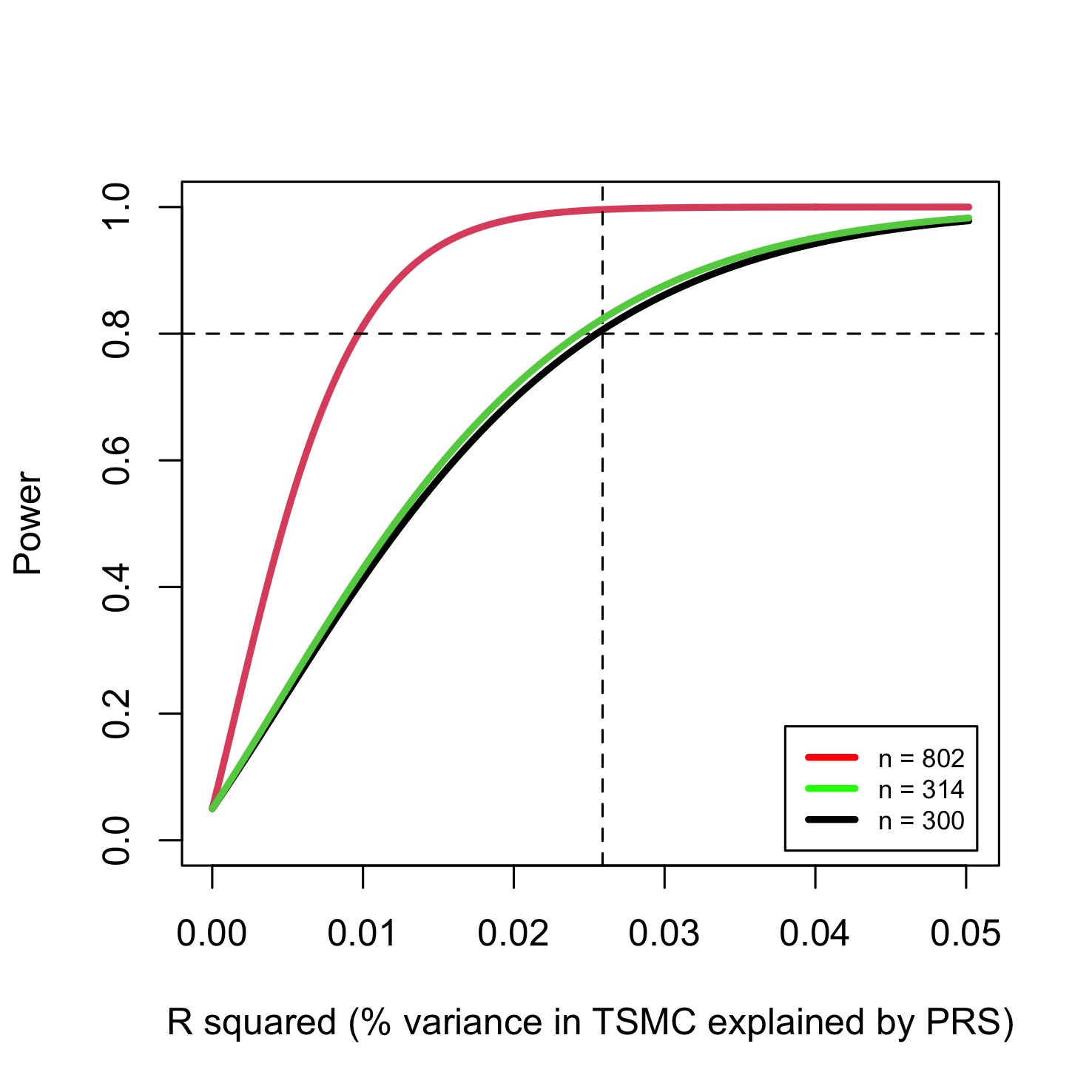
**

**Figure S1.** Power as a function of proportion of variance in TSMC explained by PRS for various sample sizes. Power was calculated based on testing the association between PRS and TSMC for various proportions of variance and sample sizes at a type I error rate of 5%. The vertical dashed line represents the effect size for the rs2588809-TSMC association from Zhu et al (32). We have at least 80% power (horizontal dashed line) to detect an association at (or greater than) the previously reported magnitude with sample size greater than 300.

**
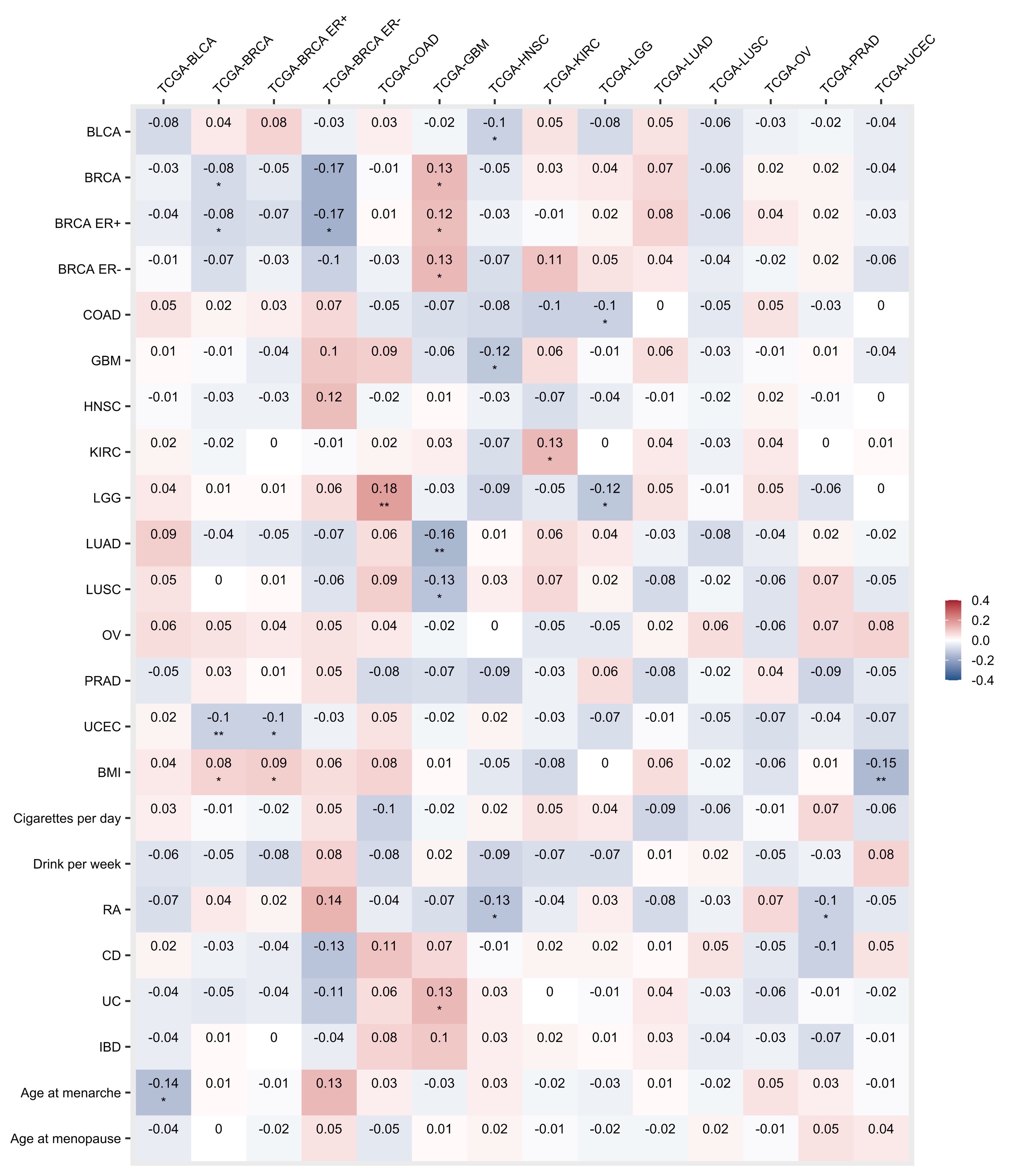
**

*** p < 0.05/322

** p < 0.01

* p < 0.05

**Figure S2.** Correlations between germline PRS and age at cancer diagnosis for each cancer type. Number in each cell and the cell color represent the Spearman correlation (ρ) between germline PRS of cancers and non-cancer traits (y-axis) and age at cancer diagnosis in a cancer type (x-axis). Corrections passed the Bonferroni threshold (*p* < 0.05/322 = 1.55 × 10^-4^) are marked with triple asterisk (***), correlations with *p* < 0.01 are marked with double asterisk (**), and correlations with *p* < 0.05 are marked with single asterisk (*).

**
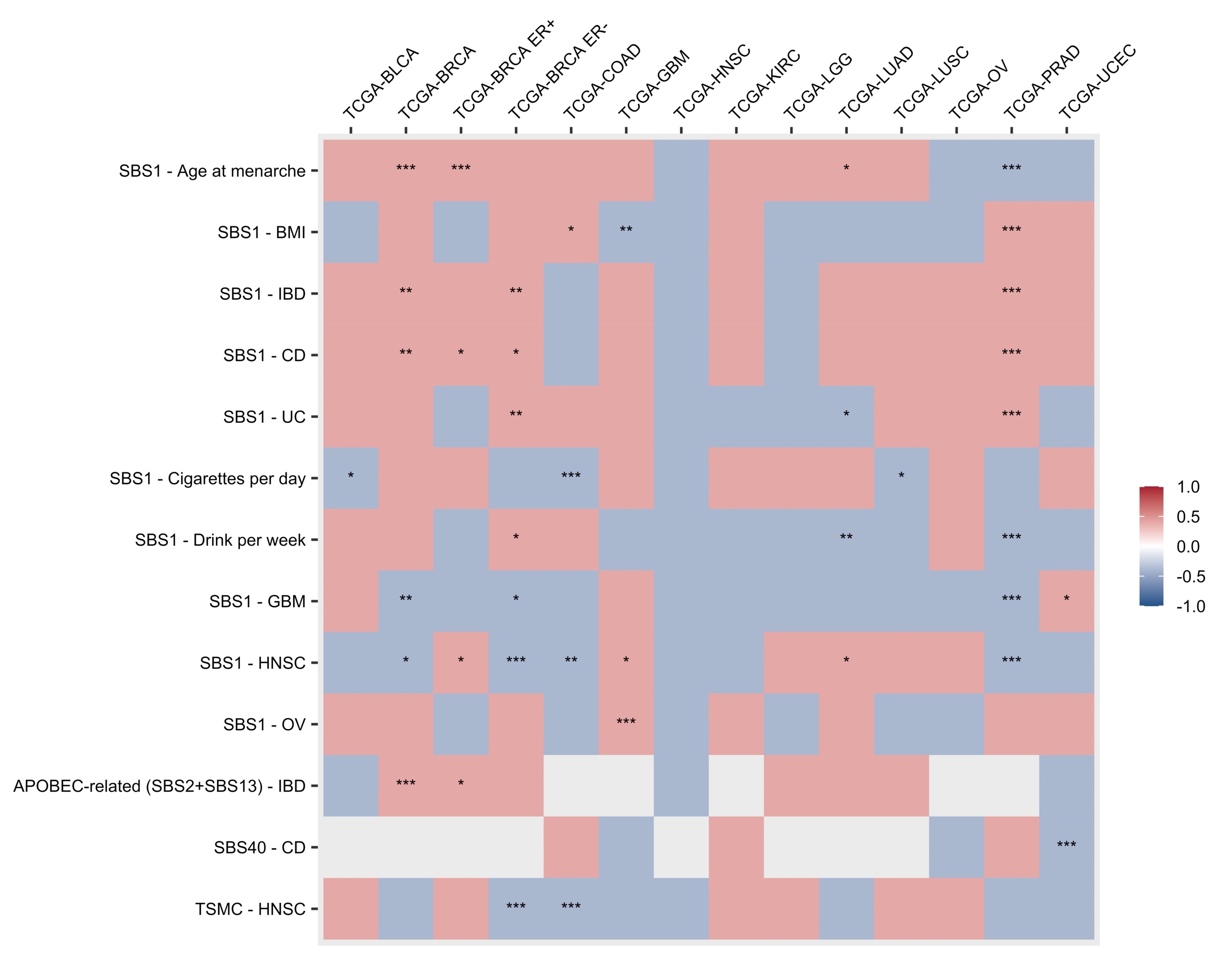
**

*** p < 0.05/1587

** p < 0.01

* p < 0.05

**Figure S3.** Significant associations between SBS signatures (or TSMC) and germline PRS across cancers. The cell color represents the direction of association between the number of somatic mutations of a SBS signature or TSMC and a germline PRS (y-axis) in a cancer type (x-axis): red = positive association; blue = inverse association. Associations passed the Bonferroni threshold (*p* < 0.05/1587 = 3.15 × 10^-5^) are marked with triple asterisk (***), associations with *p* < 0.01 are marked with double asterisk (**), and associations with *p* < 0.05 are marked with single asterisk (*).


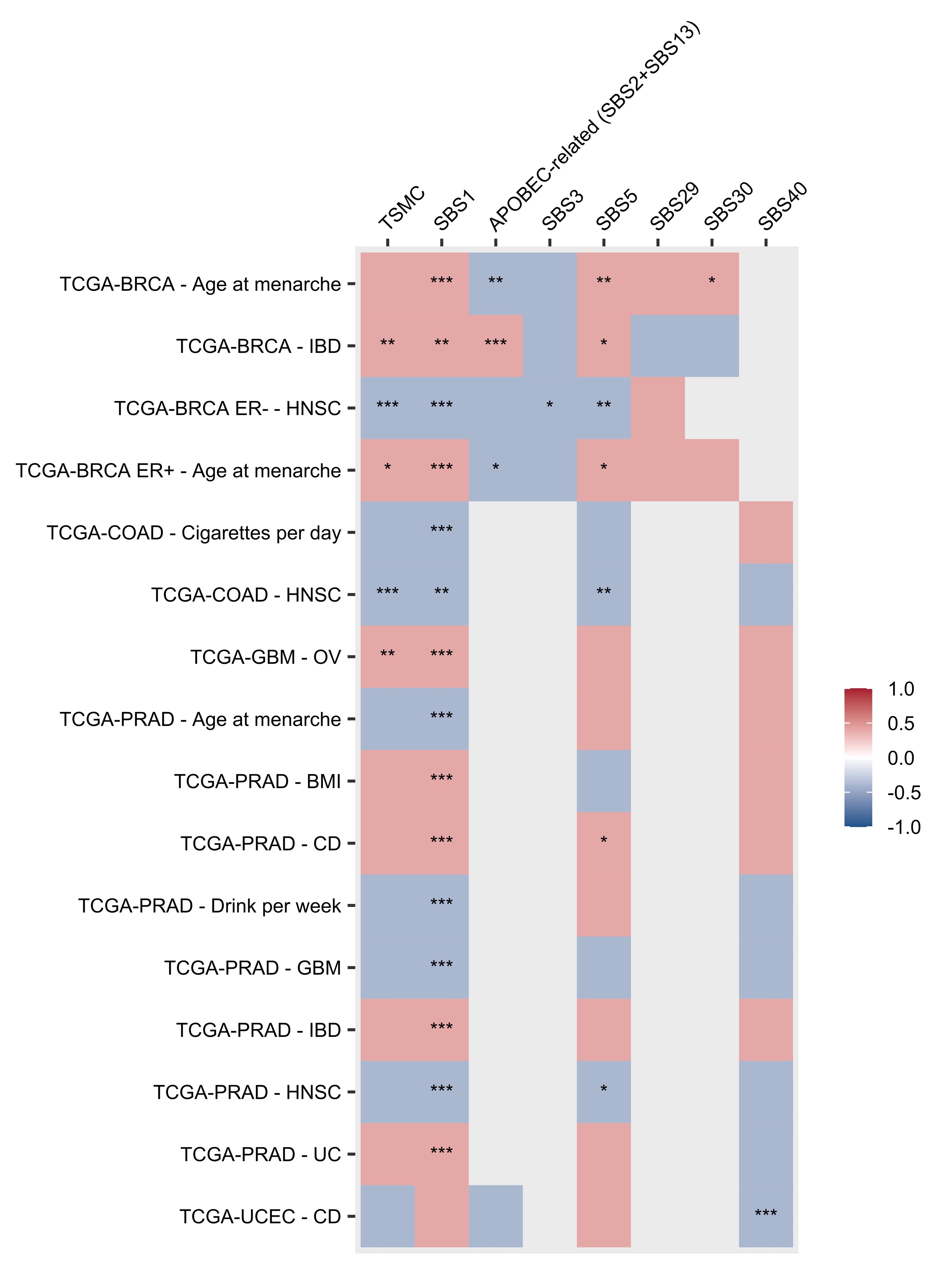


**Figure S4.** Significant associations between somatic mutation counts and germline PRS across SBS signatures and TSMC. The cell color represents the direction of association between the number of somatic mutations of a SBS signature or TSMC (x-axis) and a germline PRS in a cancer type (y-axis): red = positive association; blue = inverse association. Associations passed the Bonferroni threshold (*p* < 0.05/1587 = 3.15 × 10^-5^) are marked with triple asterisk (***), associations with *p* < 0.01 are marked with double asterisk (**), and associations with *p* < 0.05 are marked with single asterisk (*).

**References**

1. Fritsche LG, Patil S, Beesley LJ, VandeHaar P, Salvatore M, Ma Y, et al. Cancer PRSweb: An Online Repository with Polygenic Risk Scores for Major Cancer Traits and Their Evaluation in Two Independent Biobanks. Am J Hum Genet. 2020;107(5):815-36.

2. Figueroa JD, Ye Y, Siddiq A, Garcia-Closas M, Chatterjee N, Prokunina-Olsson L, et al. Genome-wide association study identifies multiple loci associated with bladder cancer risk. Hum Mol Genet. 2014;23(5):1387-98.

3. Kiemeney LA, Sulem P, Besenbacher S, Vermeulen SH, Sigurdsson A, Thorleifsson G, et al. A sequence variant at 4p16.3 confers susceptibility to urinary bladder cancer. Nat Genet. 2010;42(5):415-9.

4. Kiemeney LA, Thorlacius S, Sulem P, Geller F, Aben KK, Stacey SN, et al. Sequence variant on 8q24 confers susceptibility to urinary bladder cancer. Nat Genet. 2008;40(11):1307-12.

5. Rafnar T, Sulem P, Thorleifsson G, Vermeulen SH, Helgason H, Saemundsdottir J, et al. Genome-wide association study yields variants at 20p12.2 that associate with urinary bladder cancer. Hum Mol Genet. 2014;23(20):5545-57.

6. Rafnar T, Vermeulen SH, Sulem P, Thorleifsson G, Aben KK, Witjes JA, et al. European genome-wide association study identifies SLC14A1 as a new urinary bladder cancer susceptibility gene. Hum Mol Genet. 2011;20(21):4268-81.

7. Rothman N, Garcia-Closas M, Chatterjee N, Malats N, Wu X, Figueroa JD, et al. A multi-stage genome-wide association study of bladder cancer identifies multiple susceptibility loci. Nat Genet. 2010;42(11):978-84.

8. Wu X, Ye Y, Kiemeney LA, Sulem P, Rafnar T, Matullo G, et al. Genetic variation in the prostate stem cell antigen gene PSCA confers susceptibility to urinary bladder cancer. Nat Genet. 2009;41(9):991-5.

9. Mavaddat N, Michailidou K, Dennis J, Lush M, Fachal L, Lee A, et al. Polygenic Risk Scores for Prediction of Breast Cancer and Breast Cancer Subtypes. Am J Hum Genet. 2019;104(1):21-34.

10. Huyghe JR, Bien SA, Harrison TA, Kang HM, Chen S, Schmit SL, et al. Discovery of common and rare genetic risk variants for colorectal cancer. Nat Genet. 2019;51(1):76-87.

11. Kinnersley B, Labussiere M, Holroyd A, Di Stefano AL, Broderick P, Vijayakrishnan J, et al. Genome-wide association study identifies multiple susceptibility loci for glioma. Nat Commun. 2015;6:8559.

12. Melin BS, Barnholtz-Sloan JS, Wrensch MR, Johansen C, Il'yasova D, Kinnersley B, et al. Genome-wide association study of glioma subtypes identifies specific differences in genetic susceptibility to glioblastoma and non-glioblastoma tumors. Nat Genet. 2017;49(5):789-94.

13. Graff RE, Cavazos TB, Thai KK, Kachuri L, Rashkin SR, Hoffman JD, et al. Cross-cancer evaluation of polygenic risk scores for 16 cancer types in two large cohorts. Nat Commun. 2021;12(1):970.

14. Lambert SA, Gil L, Jupp S, Ritchie SC, Xu Y, Buniello A, et al. The Polygenic Score Catalog as an open database for reproducibility and systematic evaluation. Nat Genet. 2021;53(4):420-5.

15. Buniello A, MacArthur JAL, Cerezo M, Harris LW, Hayhurst J, Malangone C, et al. The NHGRI-EBI GWAS Catalog of published genome-wide association studies, targeted arrays and summary statistics 2019. Nucleic Acids Res. 2019;47(D1):D1005-D12.

16. Landi MT, Chatterjee N, Yu K, Goldin LR, Goldstein AM, Rotunno M, et al. A genome-wide association study of lung cancer identifies a region of chromosome 5p15 associated with risk for adenocarcinoma. Am J Hum Genet. 2009;85(5):679-91.

17. McKay JD, Hung RJ, Han Y, Zong X, Carreras-Torres R, Christiani DC, et al. Large-scale association analysis identifies new lung cancer susceptibility loci and heterogeneity in genetic susceptibility across histological subtypes. Nat Genet. 2017;49(7):1126-32.

18. Phelan CM, Kuchenbaecker KB, Tyrer JP, Kar SP, Lawrenson K, Winham SJ, et al. Identification of 12 new susceptibility loci for different histotypes of epithelial ovarian cancer. Nat Genet. 2017;49(5):680-91.

19. Schumacher FR, Al Olama AA, Berndt SI, Benlloch S, Ahmed M, Saunders EJ, et al. Association analyses of more than 140,000 men identify 63 new prostate cancer susceptibility loci. Nat Genet. 2018;50(7):928-36.

20. Cheng TH, Thompson DJ, O'Mara TA, Painter JN, Glubb DM, Flach S, et al. Five endometrial cancer risk loci identified through genome-wide association analysis. Nat Genet. 2016;48(6):667-74.

21. O'Mara TA, Glubb DM, Amant F, Annibali D, Ashton K, Attia J, et al. Identification of nine new susceptibility loci for endometrial cancer. Nat Commun. 2018;9(1):3166.

22. Spurdle AB, Thompson DJ, Ahmed S, Ferguson K, Healey CS, O'Mara T, et al. Genome-wide association study identifies a common variant associated with risk of endometrial cancer. Nat Genet. 2011;43(5):451-4.

23. Day FR, Thompson DJ, Helgason H, Chasman DI, Finucane H, Sulem P, et al. Genomic analyses identify hundreds of variants associated with age at menarche and support a role for puberty timing in cancer risk. Nat Genet. 2017;49(6):834-41.

24. Pasquale LR, Aschard H, Kang JH, Bailey JN, Lindstrom S, Chasman DI, et al. Age at natural menopause genetic risk score in relation to age at natural menopause and primary open-angle glaucoma in a US-based sample. Menopause. 2017;24(2):150-6.

25. Gao C, Patel CJ, Michailidou K, Peters U, Gong J, Schildkraut J, et al. Mendelian randomization study of adiposity-related traits and risk of breast, ovarian, prostate, lung and colorectal cancer. Int J Epidemiol. 2016;45(3):896-908.

26. Khera AV, Chaffin M, Wade KH, Zahid S, Brancale J, Xia R, et al. Polygenic Prediction of Weight and Obesity Trajectories from Birth to Adulthood. Cell. 2019;177(3):587-96 e9.

27. Liu M, Jiang Y, Wedow R, Li Y, Brazel DM, Chen F, et al. Association studies of up to 1.2 million individuals yield new insights into the genetic etiology of tobacco and alcohol use. Nat Genet. 2019;51(2):237-44.

28. Barr PB, Ksinan A, Su J, Johnson EC, Meyers JL, Wetherill L, et al. Using polygenic scores for identifying individuals at increased risk of substance use disorders in clinical and population samples. Transl Psychiatry. 2020;10(1):196.

29. Liu JZ, van Sommeren S, Huang H, Ng SC, Alberts R, Takahashi A, et al. Association analyses identify 38 susceptibility loci for inflammatory bowel disease and highlight shared genetic risk across populations. Nat Genet. 2015;47(9):979-86.

30. Khera AV, Chaffin M, Aragam KG, Haas ME, Roselli C, Choi SH, et al. Genome-wide polygenic scores for common diseases identify individuals with risk equivalent to monogenic mutations. Nat Genet. 2018;50(9):1219-24.

31. Okada Y, Wu D, Trynka G, Raj T, Terao C, Ikari K, et al. Genetics of rheumatoid arthritis contributes to biology and drug discovery. Nature. 2014;506(7488):376-81.

32. Zhu B, Mukherjee A, Machiela MJ, Song L, Hua X, Shi J, et al. An investigation of the association of genetic susceptibility risk with somatic mutation burden in breast cancer. Br J Cancer. 2016;115(6):752-60.
